# Supplementary material for: MMS SITL Ground Loop: Automating the Burst Data Selection Process
Source: Front Astron Space Sci. Author manuscript; Available in PMC 2021 Oct 27. (PMC8549770; doi:10.3389/fspas.2020.00054)
Supplement: Supplementary Information [file NIHMS1744223-supplement-Supplementary_Information.pdf]

Supplementary Material

1 SUPPLEMENTARY DATA

1.1 LSTM Model Description

It is difficult, even for SITLs, to classify any single data point as belonging to the magnetopause or not. Such classifications improve significantly, however, when put into the context of several observations before and after the current data point. The principal feature of RNNs is that outputs from units at time t are also passed as inputs to units at other time points, usually $t+1$ for unidirectional LSTMs or $t+1$ and $t-1$ for bidirectional LSTMs (which are used in the GLS MP model). The information flowing across time points makes it possible to make predictions with the context of past or future data points.

The output layer of the model, a single unit with a logistic activation function, outputs a continuous value between 0 and 1 for each data point input to the model. This continuous value is an abstraction of the model's certainty of whether or not a given data point was selected by a SITL. The SDC's pipeline for using the output selections of the GLS MP model expects a list of discrete selections, thus we need to transform the raw continuous output of the model into discrete values. We do so by simply passing the raw output of the model through a filter, giving all points that surpass a certain threshold the binary value of 1 (meaning selected) and all others the values of the 0 (meaning not selected).

Thought must be given to the value of this threshold - the value of the threshold is chosen to optimize the over-selecting datapoints (resulting in more false-positives) and the under-selecting them (resulting in more false-negatives). The MMS mission's objective is to find electron diffusion region (EDR) events associated with reconnection, a subset of which are magnetopause crossings. Although not all magnetopause crossings are EDR events, it is the in mission's best interest to select and downlink data for as many magnetopause crossings as possible. Naturally, many of these magnetopause crossings will be false-positive EDR events. However, due to memory constraints of the MMS spacecraft, we must be careful not to fill the spacecrafts' memory with false positives, risking the overwriting of true positive.

With these considerations, we have chosen a threshold of 0.5 - a value that we believe strikes a fair balance between over-selecting false-positives and under-selecting true-positives.

1.1.1 Computer Resources

Training neural networks is traditionally very computationally intensive. However, the speed of many calculations performed when training these networks can be increased by deploying the training of a model on a machine's GPU rather than its CPU. In building and training our models, we used TensorFlow Keras's CuDNNLSTM[1]layers which implement NVIDIA's CUDA Deep Neural Network Library[2]. Implementing the model with these layers thus requires the usage of a GPU. We implemented and trained our model using a Google Colab[3]- a remotely-hosted Jupyter-notebook based code editor that provides free access to GPUs (the exact GPU that the notebook is run on cannot be chosen by the user).

[1] https://www.tensorflow.org/api_docs/python/tf/compat/v1/keras/layers/CuDNNLSTM

[2] https://developer.nvidia.com/cudnn

[3] https://colab.research.google.com/

[4] https://research.google.com/colaboratory/faq.html#gpu-availability

2 SUPPLEMENTARY TABLES AND FIGURES

2.1 Statistical Study: SROI 1

Figure S1 histograms the percent overlap between individual GLS (left) or ABS (center) burst selections with selections made by the SITL, while overlap between the GLS and ABS is on the right. Panels (a-f) depict the overlap using all selections. Panels (h-m) filter the SITL segments to contain only those that were classified as MP encounters. ABS segments were filtered so that only those that overlapped with SITL MP crossings were kept. Similar plots for SROI3 and both SROI1 and SROI3 together are included in the supplemental material (Figs. S2 and S3).

2.1.1 GLS-SITL Comparison

Focusing on the GLS and SITL first, we see that if the GLS and SITL make the same selection, the SITL generally selects 100% of the GLS segment (Fig. S1a). Overall, the SITL selects 71% of all 360 GLS selections. When we take the inverse case, however, the GLS selects only 28% of SITL selections (Fig. S1d), a clear indication that the SITL is selecting a lot more than just MP crossings.

Since the GLS is trained to select only MP crossings, we next examine how many of the GLS selections overlap with intervals identified by the SITL as MP crossings. Figure S1g shows that 48% of the GLS segments were classified as MP crossings by the SITL. This can be explained partly because the SITL is aware external control factors such as telemetry restrictions and partly because some GLS segments are MP-like but are not classified as MP crossings by the SITL.

Another indicator of good model performance is that the GLS selected 76% of the 219 segments classified as MP crossings by the SITL (Fig S1j). It serves as an indicator that individual SITL classification tasks (e.g. Table 3) can be automated by ML models and that a combination of models could reduce or eliminate operations costs associated with the SITL.

2.1.2 ABS-SITL Comparison

Next, we compare the ABS to all SITL selections (Fig. S1b,e), and to only those SITL selections that were classified as MP crossings ((Fig. S1h,k). The SITL selects a larger percentage (82% vs. 72%) of ABS segments than GLS segments (Fig. S1b), but the ABS selects as few SITL segments as the GLS (Fig. S1e), again indicating that the SITL is making more selections than the ABS. Examining the MP crossings, only 19% of ABS segments were classified as MP by the SITL (Fig S1h). Conversely, only 32% of SITL-classified MP crossings were selected by the ABS (Fig S1k). So, while a majority of ABS selections are of interest to the SITL, the ABS is significantly under-selecting both in a general sense and with respect to MP crossings.

2.1.3 GLS-ABS Comparison

Lastly, we compare GLS selections with those of the ABS, and with the subset of ABS selections that overlapped with SITL MP selections. Figures S1c,f show that there is little overlap between the GLS and ABS. This is partly because of the 278 burst segments selected by the ABS, only 53 overlapped with SITL-classified MP crossings (Fig. S1l). When both the SITL and ABS select a MP crossing, the GLS does so also 79% of the time. These results indicate that, although they under-select compared to all SITL selections, the GLS and ABS are not redundant; they each make useful, complementary selections.

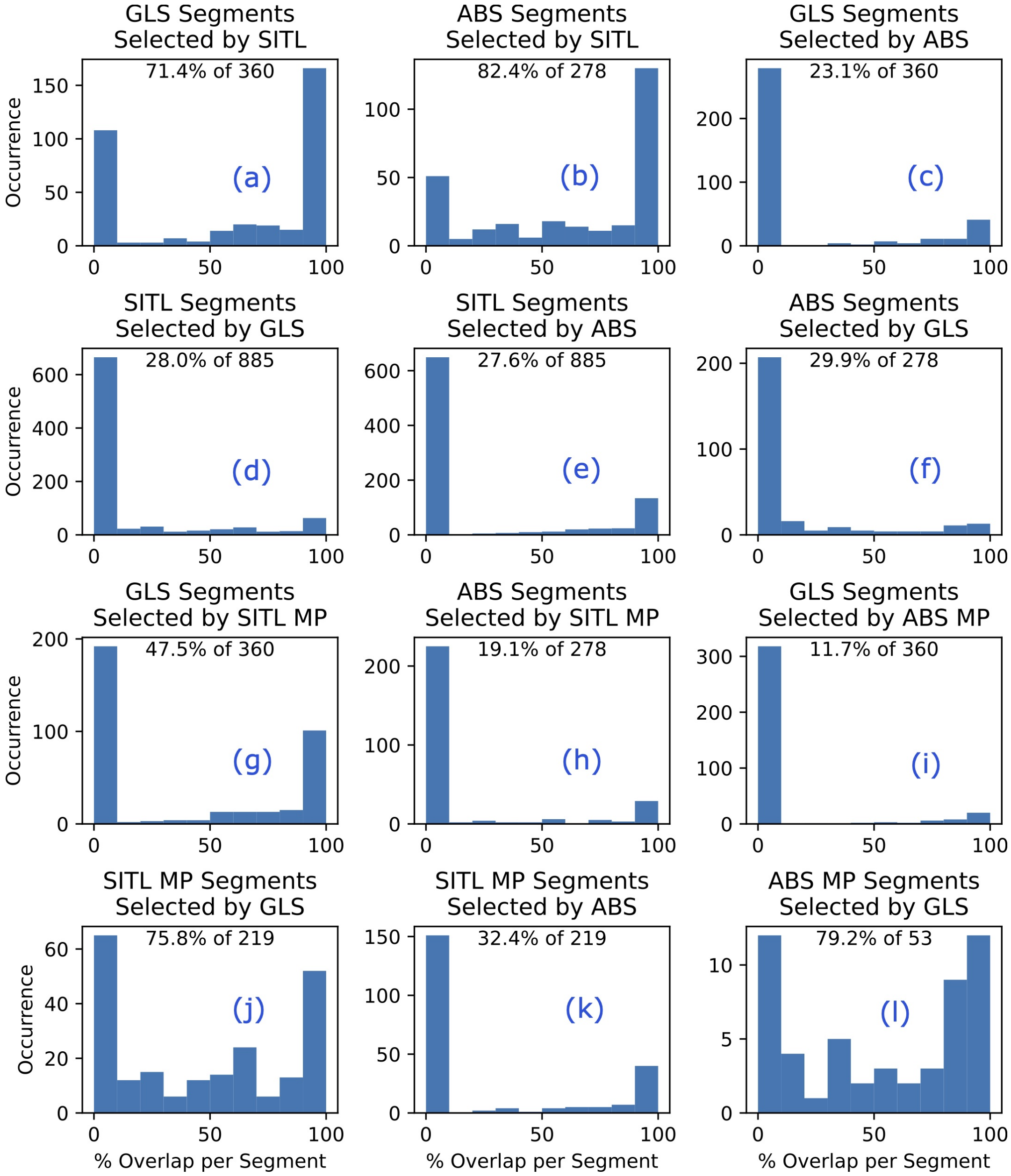

Figure S1. Comparison between of the GLS, SITL, and ABS selections showing that the GLS outperforms the ABS at selecting SITL-classified MP crossings. Columns 1-3 show overlap between the GLS and SITL, ABS and SITL, and GLS and ABS, respectively, for all selections (a-f) and only those classified as MP crossings by the SITL (g-l).

2.2 Statistical Study: SROI 3

Figure S2 shows segment overlap between the GLS, ABS, and SITL for all burst selections made in SROI3, formatted the same as Figure S1. The SITL selects a large portion of both ABS and GLS selections (Fig. S2a,b), but the ABS and GLS under-select when compared to all selections made by the SITL (Fig. S2d,e).

Extracting burst segments labeled as MP crossings by the SITL, few of either the GLS or ABS segments are classified by the SITL as MP crossings (Fig. S2g,h), similar to Figure S1g,h; however, unlike the SROI1

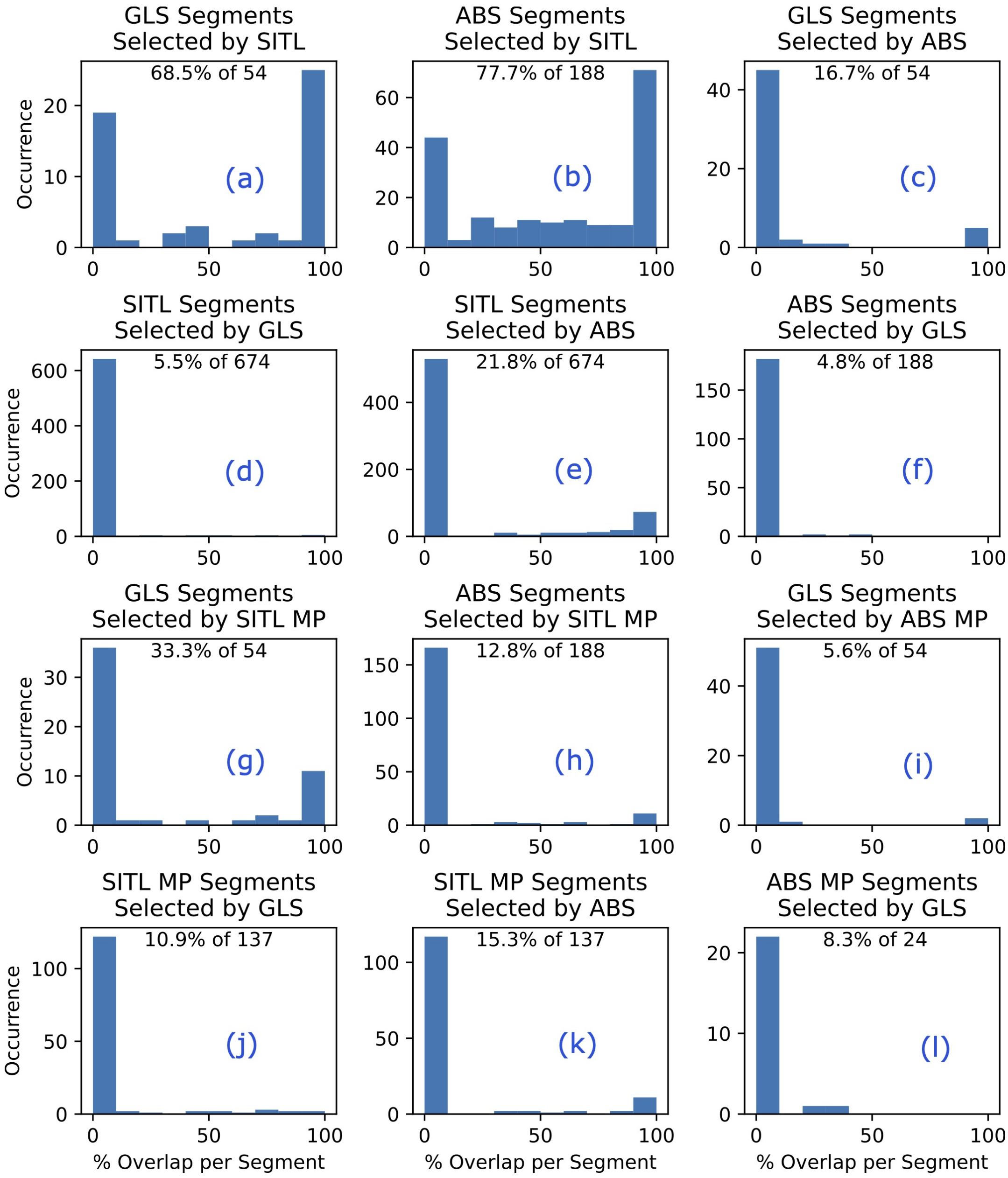

Figure S2. Overlap between burst segments made in SROI3, formatted the same as Figure S1.

selections, few SITL-classified MP crossings were selected by the GLS in SROI3 (Figure S1j). As stated in the text, the GLS is making selections of MP-like intervals but in SROI3 fails to select the MP.

As for the GLS and ABS comparison, again there is very little overlap between the two sets of selections (Fig. S2c,f,i,l), indicating that they complement one another (Fig. S2a,b).

2.3 Statistical Study: SROI 1 & 3

Figure S3 show the overlap between GLS, ABS, and SITL selections for both SROI1 and SROI3, and is formatted the same as Figure S1. Generally, the trends are the same as for Figure S1 except that the smaller number of selections made in SROI3 weight the segments with no overlap slightly heavier.

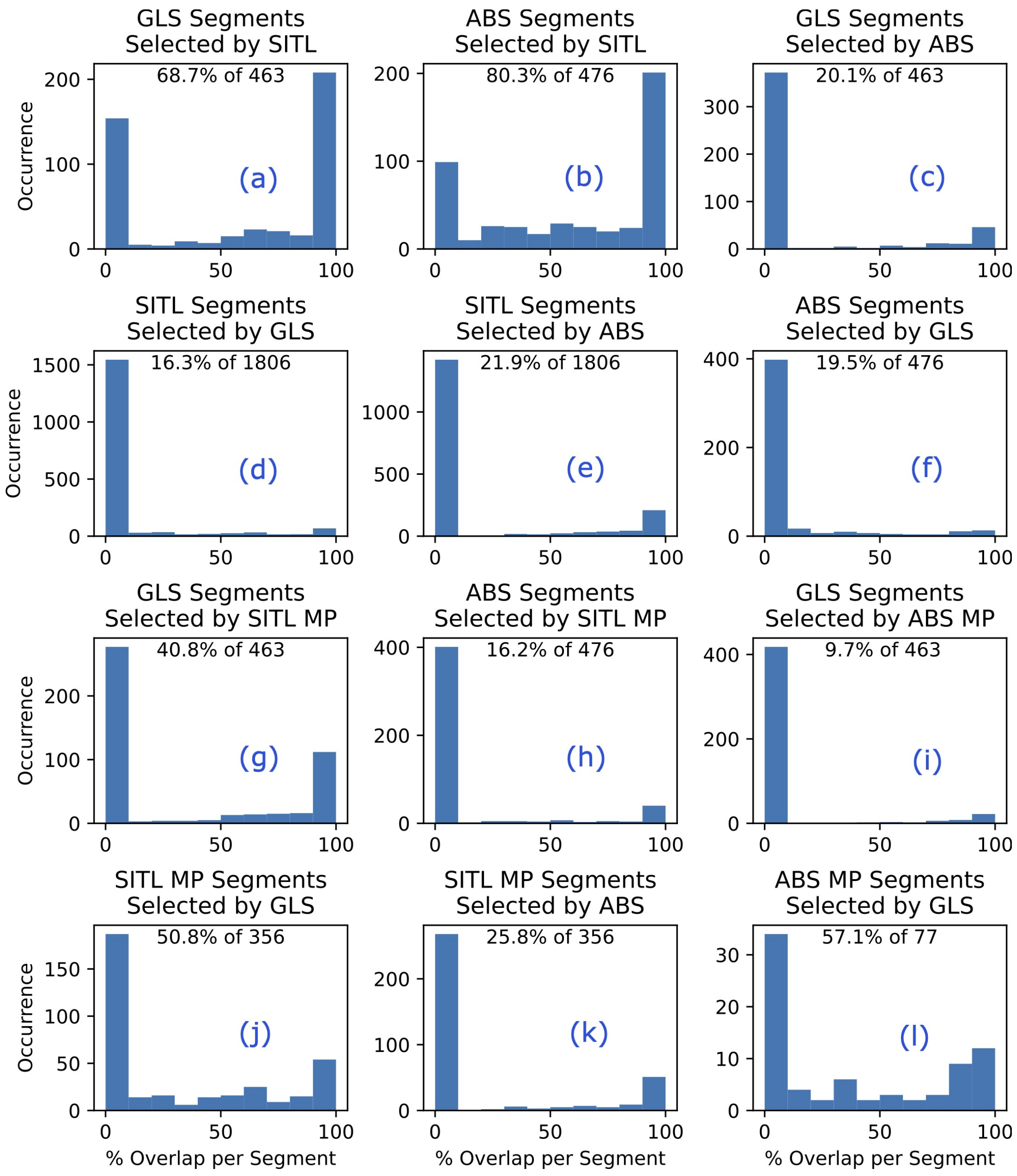

Figure S3. Overlap between burst segments made in both SROI1 and SROI3, formatted the same as Figure S1.

REFERENCES

Dungey J. Interplanetary Magnetic Field and the Auroral Zones. *Physical Review Letters* **6** (1961) 47–48. doi:10.1103/PhysRevLett.6.47.

Nagai T, Shinohara I, Fujimoto M, Matsuoka A, Saito Y, Mukai T. Construction of magnetic reconnection in the near-Earth magnetotail with Geotail. *Journal of Geophysical Research: Space Physics* **116** (2011). doi:10.1029/2010JA016283.

Nagai T, Zenitani S, Shinohara I, Nakamura R, Fujimoto M, Saito Y, et al. Ion and electron dynamics in the ion-electron decoupling region of magnetic reconnection with Geotail observations. *Journal of Geophysical Research: Space Physics* **118** (2013) 7703–7713. doi:10.1002/2013JA019135.

Oka M, Phan TD, Øieroset M, Angelopoulos V. In situ evidence of electron energization in the electron diffusion region of magnetotail reconnection. *Journal of Geophysical Research: Space Physics* **121** (2016) 1955–1968. doi:10.1002/2015JA022040.

Scudder JD, Holdaway RD, Daughton WS, Karimabadi H, Roytershteyn V, Russell CT, et al. First Resolved Observations of the Demagnetized Electron-Diffusion Region of an Astrophysical Magnetic-Reconnection Site. *Physical Review Letters* **108** (2012) 225005. doi:10.1103/PhysRevLett.108.225005.

Tang X, Cattell C, Dombeck J, Dai L, Wilson LB, Breneman A, et al. THEMIS observations of the magnetopause electron diffusion region: Large amplitude waves and heated electrons. *Geophysical Research Letters* **40** (2013) 2884–2890. doi:10.1002/grl.50565.

Mozer FS. Criteria for and statistics of electron diffusion regions associated with subsolar magnetic field reconnection. *Journal of Geophysical Research: Space Physics* **110** (2005). doi:10.1029/2005JA011258.

Scudder JD, Holdaway RD, Glassberg R, Rodriguez SL. Electron diffusion region and thermal demagnetization. *Journal of Geophysical Research* **113** (2008) 1–14. doi:10.1029/2008JA013361.

Scudder J, Daughton W. "Illuminating" electron diffusion regions of collisionless magnetic reconnection using electron agyrotropy. *Journal of Geophysical Research: Space Physics* **113** (2008). doi:10.1029/2008JA013035.

Zenitani S, Hesse M, Klimas A, Black C, Kuznetsova M. The inner structure of collisionless magnetic reconnection: The electron-frame dissipation measure and Hall fields. *Physics of Plasmas* **18** (2011) 122108. doi:10.1063/1.3662430.

Swisdak M. Quantifying gyrotropy in magnetic reconnection. *Geophysical Research Letters* **43** (2016) 43–49. doi:10.1002/2015GL066980.

Aunai N, Hesse M, Kuznetsova M. Electron nongyrotropy in the context of collisionless magnetic reconnection. *Physics of Plasmas (1994-present)* **20** (2013). doi:http://dx.doi.org/10.1063/1.4820953.

Hesse M, Aunai N, Sibeck D, Birn J. On the electron diffusion region in planar, asymmetric, systems. *Geophysical Research Letters* **41** (2014) 8673–8680. doi:10.1002/2014GL061586.

Webster JM, Burch JL, Reiff PH, Daou AG, Genestreti KJ, Graham DB, et al. Magnetospheric Multiscale Dayside Reconnection Electron Diffusion Region Events. *Journal of Geophysical Research: Space Physics* **123** (2018) 4858–4878. doi:10.1029/2018JA025245.

Burch JL, Torbert RB, Phan TD, Chen LJ, Moore TE, Ergun RE, et al. Electron-scale measurements of magnetic reconnection in space. *Science* **352** (2016). doi:10.1126/science.aaf2939.

Torbert RB, Russell CT, Magnes W, Ergun RE, Lindqvist PA, Le Contel O, et al. The FIELDS Instrument Suite on MMS: Scientific Objectives, Measurements, and Data Products. *Space Science Reviews* **199** (2016) 105–135. doi:10.1007/s11214-014-0109-8.

Phan TD, Shay MA, Eastwood JP, Angelopoulos V, Oieroset M, Oka M, et al. Establishing the Context for Reconnection Diffusion Region Encounters and Strategies for the Capture and Transmission of Diffusion Region Burst Data by MMS. *Space Science Reviews* **199** (2015) 631–650. doi:10.1007/s11214-015-0150-2.

Jian LK, Russell CT, Luhmann JG, Curtis D, Schroeder P. Burst mode trigger of STEREO in situ measurements. *AIP Conference Proceedings* **1539** (2013) 195–198. doi:10.1063/1.4811021.

Fuselier SA, Lewis WS, Schiff C, Ergun R, Burch JL, Petrinec SM, et al. Magnetospheric Multiscale Science Mission Profile and Operations. *Space Science Reviews* **199** (2016) 77–103. doi:10.1007/s11214-014-0087-x.

Baker DN, Riesberg L, Pankratz CK, Panneton RS, Giles BL, Wilder FD, et al. Magnetospheric Multiscale Instrument Suite Operations and Data System. *Space Science Reviews* **199** (2016) 545–575. doi:10.1007/s11214-014-0128-5.

Wintoft P, Wik M, Viljanen A. Solar wind driven empirical forecast models of the time derivative of the ground magnetic field. *J. Space Weather Space Clim.* **5** (2015) A7. doi:10.1051/swsc/2015008.

Boubrahimi SF, Aydin B, Martens P, Angryk R. On the prediction of >100 MeV solar energetic particle events using GOES satellite data. *2017 IEEE International Conference on Big Data (Big Data)* (2017), 2533–2542. doi:10.1109/BigData.2017.8258212.

Lundstedt H. Solar wind magnetosphere coupling: Predicted and modeled with intelligent hybrid systems. Tech. Rep. 7 (1997). doi:https://doi.org/10.1016/S0079-1946(97)00187-0.

Bhaskar A, Vichare G. Forecasting of SYMH and ASYH indices for geomagnetic storms of solar cycle 24 including St. Patrick\'{}s day, 2015 storm using NARX neural network. *J. Space Weather Space Clim.* **9** (2019) A12. doi:10.1051/swsc/2019007.

Borovsky JE. Canonical correlation analysis of the combined solar wind and geomagnetic index data sets. *Journal of Geophysical Research: Space Physics* **119** (2014) 5364–5381. doi:10.1002/2013JA019607.

Boardsen SA, Eastman TE, Sotirelis T, Green JL. An empirical model of the high-latitude magnetopause. *Journal of Geophysical Research: Space Physics* **105** (2000) 23193–23219. doi:10.1029/1998JA000143.

Jelínek K, Němeček Z, Šafránková J. A new approach to magnetopause and bow shock modeling based on automated region identification. *Journal of Geophysical Research: Space Physics* **117** (2012). doi:10.1029/2011JA017252.

da Silva D, Barrie A, Shuster J, Schiff C, Attie R, Gershman DJ, et al. Automatic Region Identification over the MMS Orbit by Partitioning n-T space (2020).

[Dataset] Olshevsky V, Khotyaintsev YV, Divin A, Delzanno GL, Anderzen S, Herman P, et al. Automated classification of plasma regions using 3D particle energy distribution (2019).

Nguyen G, Aunai N, de Welle B, Jeandet A, Fontaine D. Automatic detection of the Earth Bow Shock and Magnetopause from in-situ data with machine learning. *Annales Geophysicae Discussions* **2019** (2019) 1–22. doi:10.5194/angeo-2019-149.

Paschmann G, Baumjohann W, Sckopke N, Phan TD, Lühr H. Structure of the dayside magnetopause for low magnetic shear. *Journal of Geophysical Research: Space Physics* **98** (1993) 13409–13422. doi:10.1029/93JA00646.

Phan TD, Paschmann G. Low-latitude dayside magnetopause and boundary layer for high magnetic shear: 1. Structure and motion. *Journal of Geophysical Research: Space Physics* **101** (1996) 7801–7815. doi:10.1029/95JA03752.

Fear RC, Palmroth M, Milan SE. Seasonal and clock angle control of the location of flux transfer event signatures at the magnetopause. *Journal of Geophysical Research: Space Physics* **117** (2012). doi:10.1029/2011JA017235.

Kavosi S, Raeder J. Ubiquity of Kelvin–Helmholtz waves at Earth's magnetopause. *Nature Communications* **6** (2015) 7019. doi:10.1038/ncomms8019.

Matsui H, Farrugia CJ, Goldstein J, Torbert RB, Argall MR, Vaith H, et al. Velocity Rotation Events in the Outer Magnetosphere Near the Magnetopause. *Journal of Geophysical Research: Space Physics* **124** (2019) 4137–4156. doi:10.1029/2019JA026548.

Wing S, Johnson JR, Chaston CC, Echim M, Escoubet CP, Lavraud B, et al. Review of Solar Wind Entry into and Transport Within the Plasma Sheet. *Space Science Reviews* **184** (2014) 33–86. doi:10.1007/s11214-014-0108-9.

[Dataset] Argall MR, Small CR, Petrik M. MMS SITL Ground Loop: Data for the GLS-MP Magnetopause Model (2020a). doi:10.5281/zenodo.3884266.

[Dataset] Small CR, Argall MR, Petrik M. MMS SITL Ground Loop: Notebooks to Train and Run the GLS-MP Model (2020). doi:10.5281/zenodo.3891992.

Torbert RB, Burch JL, Phan TD, Hesse M, Argall MR, Shuster J, et al. Electron-scale dynamics of the diffusion region during symmetric magnetic reconnection in space. *Science* (2018). doi:10.1126/science.aat2998.

Zhou M, Deng XH, Zhong ZH, Pang Y, Tang RX, El-Alaoui M, et al. Observations of an Electron Diffusion Region in Symmetric Reconnection with Weak Guide Field. *The Astrophysical Journal* **870** (2019) 34. doi:10.3847/1538-4357/aaf16f.

Angelopoulos V, Cruce P, Drozdov A, Grimes EW, Hatzigeorgiu N, King DA, et al. The Space Physics Environment Data Analysis System (SPEDAS). *Space Science Reviews* **215** (2019) 9. doi:10.1007/s11214-018-0576-4.

[Dataset] Argall MR, Small CR, Petrik M. PyMMS: A Python Library for NASA's Magnetospheric Multiscale (MMS) Mission (2020b). doi:10.5281/zenodo.3840766.

Russell CT, Anderson BJ, Baumjohann W, Bromund KR, Dearborn D, Fischer D, et al. The Magnetospheric Multiscale Magnetometers. *Space Science Reviews* **199** (2014) 189–256. doi:10.1007/s11214-014-0057-3.

Ergun RE, Tucker S, Westfall J, Goodrich KA, Malaspina DM, Summers D, et al. The Axial Double Probe and Fields Signal Processing for the MMS Mission. *Space Science Reviews* **199** (2014) 167–188. doi:10.1007/s11214-014-0115-x.

Lindqvist PA, Olsson G, Torbert RB, King B, Granoff M, Rau D, et al. The Spin-Plane Double Probe Electric Field Instrument for MMS. *Space Science Reviews* **199** (2014) 137–165. doi:10.1007/s11214-014-0116-9.

Pollock C, Moore T, Jacques A, Burch J, Gliese U, Saito Y, et al. Fast Plasma Investigation for Magnetospheric Multiscale. *Space Science Reviews* **199** (2016) 331–406. doi:10.1007/s11214-016-0245-4.

Phan TD, Eastwood JP, Cassak PA, Øieroset M, Gosling JT, Gershman DJ, et al. MMS observations of electron-scale filamentary currents in the reconnection exhaust and near the X line. *Geophysical Research Letters* **43** (2016) 6060–6069. doi:10.1002/2016GL069212.

Goodfellow I, Bengio Y, Courville A. *Deep Learning*. Adaptive Computation and Machine Learning series (MIT Press) (2016).

Srivastava N, Hinton G, Krizhevsky A, Sutskever I, Salakhutdinov R. Dropout: A Simple Way to Prevent Neural Networks from Overfitting. *J. Mach. Learn. Res.* **15** (2014) 1929–1958.

Tatbul N, Lee TJ, Zdonik S, Alam M, Gottschlich J. Precision and Recall for Time Series. Bengio S, Wallach H, Larochelle H, Grauman K, Cesa-Bianchi N, Garnett R, editors, *Advances in Neural Information Processing Systems 31* (Curran Associates, Inc.) (2018), 1920–1930.

Truszkowski W, Rouff C, Bailin S, Riley M. Progressive autonomy: a method for gradually introducing autonomy into space missions. *Innovations in Systems and Software Engineering* **1** (2005) 89–99. doi:10.1007/s11334-005-0018-9.

Truszkowski WF, Hinchey MG, Rash JL, Rouff CA. Autonomous and autonomic systems: a paradigm for future space exploration missions. *IEEE Transactions on Systems, Man, and Cybernetics, Part C (Applications and Reviews)* **36** (2006) 279–291. doi:10.1109/TSMCC.2006.871600.

Truszkowski W, Hinchey M, Rash J, Rouff C. NASA's swarm missions: the challenge of building autonomous software. *IT Professional* **6** (2004) 47–52. doi:10.1109/MITP.2004.66.

Rascoff S, Humphries S. *Zillow Talk: The New Rules of Real Estate* (Grand Central Publishing) (2015).

Fennell JF, Turner DL, Lemon CL, Blake JB, Clemmons JH, Mauk BH, et al. Microinjections observed by MMS FEEPS in the dusk to midnight region. *Geophysical Research Letters* **43** (2016) 6078–6086. doi:10.1002/2016GL069207.

Kavosi S, Spence HE, Fennell JF, Turner DL, Connor HK, Raeder J. MMS/FEEPS Observations of Electron Microinjections Due to Kelvin-Helmholtz Waves and Flux Transfer Events: A Case Study. *Journal of Geophysical Research: Space Physics* **123** (2018) 5364–5378. doi:10.1029/2018JA025244.

Claudepierre S, Fennell J, Sengupta A, Kletzing C. Automated Identification of Electron Microinjections in MMS/FEEPS Measurements: Initial Results. *Earth and Space Science Open Archive* (2020) 10501706. doi:10.1002/essoar.10501706.1.

Piatt S. Large-Scale Statistical Survey of Magnetopause Reconnection. *arXiv e-prints* (2019) arXiv:1905.11359.

[Dataset] Argall MR, Small Colin R, Petrik M. MMS SITL Ground Loop: Software to Reproduce Figures and Tables (2020c). doi:10.5281/zenodo.3891944.
